# Changes of Raw Texture, Intramuscular Connective Tissue Properties and Collagen Profiles in Broiler Wooden Breast during Early Storage

**DOI:** 10.3390/foods12071530

**Published:** 2023-04-04

**Authors:** Xueshen Zhu, Eero Puolanne, Per Ertbjerg

**Affiliations:** 1 Key Laboratory of Biological Functional Molecules of Jiangsu Province, College of Life Science and Chemistry, Jiangsu Second Normal University, Nanjing 211200, China; xueshen_zhu@163.com; 2 Department of Food and Nutrition, University of Helsinki, 00014 Helsinki, Finland

**Keywords:** wooden breast, collagen profiles, intramuscular connective tissue, thermal properties, postmortem

## Abstract

A recently identified broiler myopathy known as wooden breast (WB) is predominantly found in the *pectoralis major* muscle of fast-growing broiler hybrids and is causing significant losses to the poultry industry. The aim of this study was to investigate the effects of WB syndrome on raw meat texture, purge loss and thermal properties of intramuscular connective tissue of *pectoralis major* muscle in the early postmortem period (1–3 days). Results showed that the presence of the WB muscles condition at 1 day postmortem was associated with significantly increased stiffness (27.0 N vs. 23.1 N) and significantly increased purge loss (1.8% vs. 1.0%) compared to normal breast (NB). However, on 3 days postmortem, these parameters did not differ between WB and NB groups. Insoluble and total collagen content was significantly higher in WB muscles compared to NB muscles, and the extractability of intramuscular connective tissue (IMCT) of WB was also higher (0.42% vs. 0.37%) compared to NB and remained stable in the early postmortem period. There was significantly lower protein content in the sarcoplasmic protein fraction and myofibrillar protein fraction of WB muscles compared to NB muscles (*p* < 0.05). The IMCT of these two groups showed different thermal properties, as the enthalpy of denaturation (ΔH) was significantly lower in WB muscles compared to NB muscles. The WB syndrome had a great effect on the texture and connective tissue properties of the meat compared to normal muscle, with a tendency for having a lower purge loss and higher raw meat hardness.

## 1. Introduction

The increasing demand for chicken meat has led the poultry industry over the past 50 years to focus on high-energy diets and intensive selection for genotypes that exhibit faster growth and higher breast yields. However, the breast muscle of fast-growing chickens is associated with an increased number of giant fibers, which are typically three to five times larger in cross-sectional area than those of slower-growing chickens [1]. The incidence of many *pectoral* meat abnormalities has increased dramatically over time. White striping, deep *pectoral* disease also known as Oregon disease, PSE-like breast meat and poor meat cohesion are some examples of the various observed defects [2,3,4]. Recently, wooden breast (WB) myopathy has become a growing concern as affected fillets have an unsightly appearance, and it has been found in many countries around the world [5].WB is generally characterized by muscle fiber necrosis, inflammatory cell accumulation and fibrosis [6,7]. The pathophysiology of this condition remains unknown, but oxidative stress in the poorly vascularized breast muscle has been anticipated to be the causative factor [5]. In contrast to normal meat, WB defects can be classified by palpation, for example, on the slaughter line, based on a harder texture [6]. Although WB-affected cuts of meat are edible without any health risk, they are not readily accepted by consumers due to outward appearance and texture problems, thus leading to losses in the poultry industry and potential poultry welfare issues. Wooden breast myopathy is associated with impairment of gait scores and may also cause welfare problems [8]. Therefore, WB is a concern for the poultry industry, as this myopathy can cause an unpleasant consumer experience and thus affect consumer acceptance. There is much evidence regarding a plausible etiology. Recent RNA-seq analysis studies suggest that local hypoxia, oxidative stress, higher intracellular calcium levels, and muscle fiber type conversion associated with modern fast-growing broilers may be associated with the development of these myopathies [9]. The occurrence of defects also seems to be influenced by location in the muscle: the thickest part of the breast muscle, the cranial portion, may be susceptible to hyperextension or ischemia, leading to tissue damage and repair responses due to impaired blood supply [2].

A recent study has shown a progressive course of this disease with acute vasculitis confined to small caliber veins, lipid infiltration and deposition, and an early stage of fibrosis followed by a chronic fibrotic stage [6]. Microscopically, there is muscle degeneration with regeneration and accumulation of loose connective tissue in the muscle as well as thickening of epimysial membrane [7]. Regardless of storage temperature, consistent results including higher hardness and cook loss in WB muscles compared to NB were found in early postmortem. However, the underlying mechanisms seemed to be an open topic [5]. Nonetheless, connective tissue is a minor element of meat, its contribution to texture is important, but not well understood in WB. The objective of this study was to investigate changes in texture, intramuscular connective tissue properties and collagen profile of broiler wooden breast *pectoral* muscle during early storage.

## 2. Materials and Methods

### 2.1. Sample Collection and Chemicals

**Figure 1:**
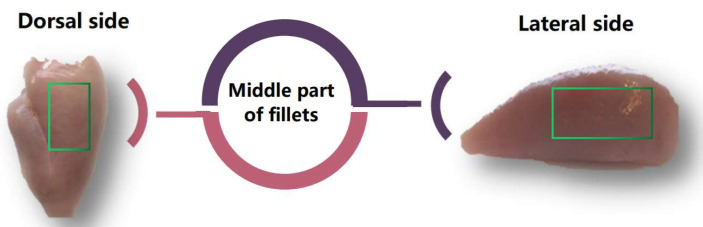
Sampling position diagram.

A total of 12 31-day-old broilers (*Ross 308*) were collected at a commercial slaughterhouse (Saarioinen Plc, Sahalahti, Finland). Muscles were sampled on the cutting line three hours postmortem, in one day. The selection of fillets was based on visual appearance and palpation of the *pectoralis major* muscle in fillets showing the wooden breast (WB) or normal breast (NB) condition; *pectoralis major* muscles exhibiting diffuse hard areas with color defects and petechiae were noted as WB. The WB status used here can be regarded as severe WB according to commonly used grading [10,11]. In contrast, fillets with soft and elastic tissue and uniform color were rated as NB. Immediately after the selection, the breasts were placed in polyethylene bags on ice and transported to the University of Helsinki, Finland, Department of Food Science and Nutrition, Meat Laboratory. The middle parts of the fillets were excised (Figure 1) and stored at 4 °C for 72 h. A total of 6 pieces of wooden breast meat (WB) and 6 pieces of normal breast meat (NB) were used, with the average weight of 413.4 ± 29.7 g and 292.2 ± 46.8 g, respectively. All chemical reagents were chemically pure.

### 2.2. Compression Test

Compression tests were performed on a TA-XT2i texture analyzer (Stable Micro System Ltd., Godalming, UK). In this study, the measurement unit was modified mainly according to Soglia et al. [12], where muscle samples were deformed transversely in only one direction, which means that the compressed meat strips can only extend longitudinally. Compression forces were measured on raw chicken samples. Each chicken fillet from the inner layer was cut into three 1 × 1 × 3 cm strips with muscle fibers parallel to the longitudinal direction. The test was performed by compressing the sample to 80% of its initial height with a trigger force of 5 g at a speed of 50 mm/min. The highest compression values were filtered out from all records. The average of three measurements was recorded for each of the three strips of one fillet.

### 2.3. Purge Loss Measurements

For purge loss measurements, about 20 g of muscle sample was kept in a sealed polyethylene package at 4 °C. After the bags were opened, the exudation on the surface of muscles was removed with filter paper. The weight of the muscle samples was recorded again, and the weight loss, expressed as a percentage of initial weight, was regarded as purge loss. The purge loss was calculated from the average of three replicates for each muscle.

### 2.4. Collagen Profiles Measurements

The method for analysis of insoluble collagen and total collagen content was adopted from Latorre et al. [13]. Muscles were first minced using a blender (Blendtec, Orem, UT, USA), then samples in triplicates (2.5 g) were weighed and transferred into the digestion tubes. After addition of 30 mL of 6 M sulphuric acid, the flask was covered with a watch glass. The samples were hydrolyzed in a digester (Tecator Digestion System 20-1015, Tecator, Inc., Herndon, VA, USA) at 110 °C for 16 h. The hydrolyzed samples were diluted in 100 mL milliQ water and filtered using Whatman No 1 filter paper. The filtered samples were further diluted in 50 mL milliQ water and neutralized with 6 M NaOH. The hydroxyproline in the samples was oxidized by chloramine-T in the prepared aqueous buffer solution, followed by colorimetric reaction with the 4-(Dimethylamino)benzaldehyde. The samples were then incubated at 60 °C for 30 min and after cooling and letting the samples set for 25 min, absorbance was measured at λ = 560 nm using a spectrophotometer (Ordior Shimadzu UV Spectrophotometer, Shimadzu Corporation, Kyoto, Japan). The hydroxyproline content was determined against a standard calibration curve prepared in a similar manner. The total collagen content was determined from hydroxyproline content by using a conversion factor of 7.25 and finally expressed as a percentage.

In order to measure the insoluble collagen content, the minced muscle samples were first kept in a water bath at 77 °C for 65 min, then centrifuged at 5800 rpm for 10 min. The precipitates were taken as the insoluble part. The average of three measurements of total and insoluble collagen content of each sample was recorded.

### 2.5. Filter Residue and Thermal Properties of IMCT Analysis

Filter residues of intramuscular connective tissue were analyzed as described by Chang et al. [14] with some modifications. Forty grams wet weight of raw breast was first cut into 0.5 cm^3^ cubes and was homogenized in 50 mL of ice-cold water for 30 s at 3000 rpm using a IKA-T25 homogenizer (Labortechnik, Staufen, Germany). The homogenate was filtered through a metal sieve (1 mm^2^ perforations), and the material retained on the filter was re-homogenized in 50 mL of CaCl_2_ and re-filtered. The process was repeated three times. The material retained on the filter, designated as filtering residues, was freeze-dried (Alpha 21.2, Christ, Germany) until a constant weight was reached. The contents of filtering residues were calculated as a percentage of the initial wet sample weight. Filter residues were mainly composed of intramuscular connective tissue (IMCT).

Thermal properties of intramuscular connective tissue were analyzed by differential scanning calorimetry (DSC) to determine the onset, peak and end temperatures (To, Tp, Te, respectively); and the enthalpy (ΔH) of thermal denaturation of intramuscular connective tissue [15]. Briefly, 10% (*w*/*w*) of milliQ water was added to the freeze-dried samples of IMCT, which were then kept overnight to equilibrate the water distribution. The ΔH, the extra energy that was needed to maintain a constant increase of temperature during DSC analysis when collagen in the intramuscular connective tissue sample contracted, was expressed in J/g. Approximately 10 mg was placed in a 40 µL aluminum DSC sample pan and the pans were hermetically sealed. The samples were heated from 10 to 95 °C at a heating rate of 5 °C/min using a TA 4000, DSC 30 (Mettler Toledo, Greifensee, Switzerland). The instrument was calibrated before use. An empty sample pan was used as a reference. After the DSC analysis, the lid of each sample pan was pierced, the sample was then dried overnight at 103 °C to measure the weight.

### 2.6. Protein Extraction Analysis and Thiol Groups Content Measurements

Samples for the determination of protein extraction and thiol groups content were taken at 1 d postmortem. These samples were immediately frozen in liquid nitrogen and stored at −80 °C until analysis. Sarcoplasmic and myofibrillar protein fractions were extracted according to Zhu et al. [16]. Exactly 1.00 g of frozen muscle samples was added to 10 mL of rigor buffer containing 0.075 M KCl, 0.010 M KH_2_PO_4_, 0.002 M MgCl_2_, 0.002 M EGTA, pH 7.0 and homogenized using a IKA-T25 homogenizer (Labortechnik, Staufen, Germany) at 13,500 rpm for 20 s. The homogenate was centrifuged at 10,000× *g* for 10 min at 4 °C, and the supernatant was decanted and saved as the sarcoplasmic protein fraction. The homogenization of the pellet in 20 mL fresh rigor buffer and centrifugation was repeated 3 times to extract the sarcoplasmic proteins and to obtain the myofibrillar protein fraction pellet. The final pellet was then homogenized in 20 mL rigor buffer to obtain the suspension of myofibrillar protein fraction. The protein content of the sarcoplasmic protein fraction and myofibrillar protein fraction was determined using the BCA protein kit (Merck KgaA, Darmstadt, Germany) and calculated as the average of three replicates of each muscle sample. Protein thiol groups were then determined according to Bao et al. with minor modification [17]. Briefly, one ml of sarcoplasmic and myofibrillar protein fraction extracted as above was homogenized with 10 mL 5% SDS in 0.1 M Tris–HCl (pH 8.0) at 13,500 rpm for 30 s, separately. The homogenates were heated in a water bath at 80 °C for 30 min. After cooling, the homogenates were filtered through filter paper (Whatman 40, GE Healthcare). The protein concentration of the filtrate was determined by reading absorbance at 280 nm. Thiol groups were measured by mixing 0.5 mL filtrate, 2 mL of 0.1 M Tris–HCl (pH 8.0) and 0.5 mL 10 mM 5,5’-Dithiobis (2-nitrobenzoic acid) in 0.1 M Tris–HCl (pH 8.0). The mixture was incubated in the dark at room temperature for 30 min. Absorbance at 412 nm was recorded and the content of thiol groups was calculated and expressed as nmol/mg protein.

### 2.7. Statistical Analyses

Data were analyzed using the SPSS^®^ Statistics Version 21 package (IBM, Chicago, IL, USA). Duncan’s multiple range test for statistical analysis was performed with SPSS and graphical representations were performed with Excel 2010 (Microsoft, Redmond, DC, USA).

## 3. Results and Discussion

### 3.1. Compression Values and Purge Loss

**Figure 2:**
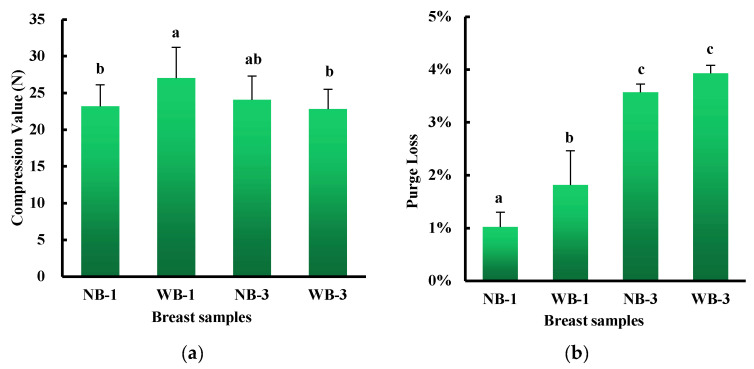
Compression value (**a**), purge loss (**b**) of wooden breast (WB) and normal breast (NB) muscles of broilers at day 1 and day 3 postmortem. Means without a common superscript (abc) differ; *p* < 0.05.

As expected, on day 1, raw breast meat within WB had higher compression values than normal breast (Figure 2a, *p* < 0.05); however, on postmortem day 3, there was no difference in compression values between these two groups. These results suggest a greater tendency for WB compression values to decrease during storage compared to normal compression values. Petracci et al. [4] reported that the increase in overall connective tissue was detrimental to the protein content of WB meat, which was also found to be lower than the content in normal meat. Increased compression values in the caudal *pectoralis major* affected by WB abnormalities have been previously reported [18]. Furthermore, Soglia et al. found a progressive tendency of softening of both superficial and deep layers of raw WB samples from 10 to 72 h postmortem [12]. It should be noted that the increase in interstitial connective tissue seen in WB samples leading to fibrosis [6], as well as the increased deposition of extracellular matrix [7], likely had an impact on the hardness of the raw meat. The fibrosis in wooden breast-affected muscle is characterized by the replacement of muscle fibers by extracellular matrix proteins, particularly fibril-forming collagen [19].

Along with higher hardness, the purge loss of WB was also significantly higher than that of NB on 1 day postmortem (Figure 2b), indicating that WB had much lower water holding capacity than NB on 1 day postmortem. However, no difference was found in the total purge loss between the two groups after 3 days of refrigeration. Similarly, Mudalal et al. reported that the presence of WB impaired not only the appearance of fillets but also the quality of raw and cured meat, mainly by reducing the water holding capacity. Compared to NB, WB showed higher compression values and cooking losses in raw meat, while there was no difference in cooked meat shear force [18]. More recently, Tasoniero et al. [20] investigated the role of the physico-chemical state of myowater on the development of hardness in WB by NMR relaxometry and reported that water redistribution occurred over time during storage, as evidenced by the increasing trend in T_21_ population. The cranial/superficial portion of the breast exhibited the highest amount of the extramyofibrillar water population (T_22_) and the texture of this part of the muscle was stiffer than the deeper layers. It may be noted that although water loss was higher in WB-affected samples on one day postmortem, it remained in the range of values observed normally at 24 h postmortem.

### 3.2. Collagen Profile

**Figure 3:**
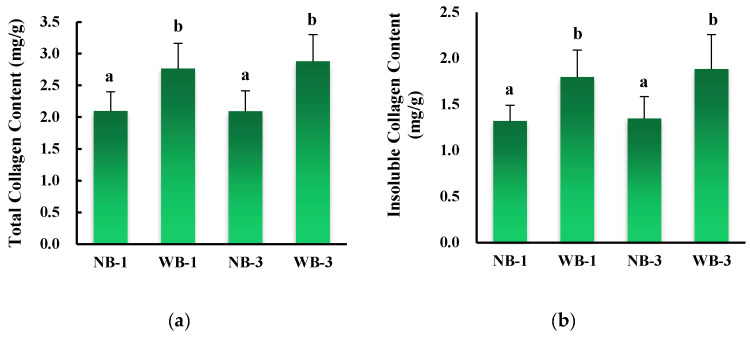
Total collagen (**a**) and insoluble collagen content (**b**) in wooden breast (WB) and normal breast (NB) muscles of broilers at day 1 and day 3 postmortem. Means without a common superscript (ab) differ; *p* < 0.05.

The result of collagen content analysis in our study is presented in Figure 3. Overall, the total amount of collagen in WB affected muscles was higher than normal on postmortem days 1 and 3 (*p* < 0.05). These results coincide with the findings of Soglia et al. [12]. The middle part (ventral area) of the WB samples had higher levels of insoluble collagen compared to normal samples (Figure 3b). Recent literature has also shown that insoluble, soluble and total collagen were also higher in wooden breast heavy fillets than in normal fillets at 9 weeks of age [5]. In general, WB shows muscle degradation conditions and a relatively high collagen content, which was also demonstrated by microscopy studies showing large areas of connective tissue in WB muscle [21]. Although this is very clear in the previous literature, it is worth reiterating. Compression measurements of raw meat generally show good correlations with collagen content and collagen properties of different muscles [22], and within the *longissimus* muscle of beef the collagen properties show correlation with raw meat texture [23]. Moreover, large proteoglycans interact with hyaluronic acid to form larger aggregates that provide swelling pressure as well as matrix elasticity, ultimately giving the tissue stiffness [24]. Regarding the intramuscular connective tissue (IMCT) properties in this study, the results showed that the interior of WB showed the highest amount of insoluble and total collagen compared to normal samples, which may be related to the increase in tissue stiffness and the decrease in fleshiness.

### 3.3. Filter Residue and Thermal Properties of Intramuscular Connective Tissue

**Figure 4:**
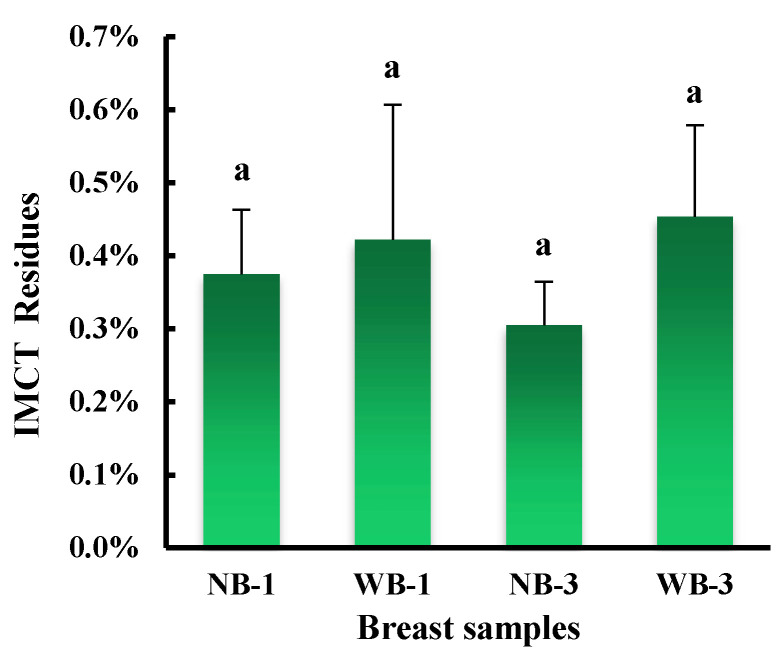
Intramuscular connective tissue (IMCT) residues after extraction and filtration in wooden breast (WB) and normal breast (NB) muscles of broilers at day 1 and day 3 postmortem. Means without a common superscript differ, *p* < 0.05.

Thermal properties are an important parameter of connective tissue. Compared to NB, more IMCT was extracted from WB, not only on day 1 but also on day 3 postmortem (Figure 4). The DSC results showed no significant differences at the beginning and peak temperatures; however, the enthalpy of denaturation (ΔH) was significantly lower for WB compared to NB (Table 1) and the endset temperature of WB was significantly lower compared to NB at 3 days postmortem. It should be mentioned that there was a slight downward trend in ΔH for WB and a subtle downward trend in end temperature from day 1 to day 3. According to Kopp et al. (1990), collagen in IMCT showed a decrease in ΔH with increasing collagen cross-linking in dried samples of corrugated muscle, suggesting that the result of hydrophobic action corresponds to a change in stable cross-linked collagen fibers [25]. We speculated that an alteration involving cross-linkages in the structure of the intramuscular connective tissue of WB may explain the lower ΔH; in addition, an increased amount of cross-linkages attributed to decorin, a proteoglycan that mediates collagen crosslinking, growth factor signaling, and cell growth in WB connective tissue [26], could be another reason. SDS-PAGE patterns showed that the protein profiles of intramuscular connective tissue extracted from WB and NB muscles differed (data not shown). The thermal and mechanical stability of intramuscular connective tissue is primarily related to the chemical nature of covalent intermolecular cross-linking of collagen [27]. Velleman and Clark [7] used real-time quantitative PCR analysis of WB muscle and found that the expression levels of decorin, a regulator of collagen cross-linking, correlated with differences in collagen organization. Differences in connective tissue composition may result in different thermal properties. Increased stiffness in muscle affected by WB is not only associated with increased collagen content, but also with the degree of fibrillated collagen and structural features such as fiber diameter, cross-linking, fiber density, and other structural features [28]. According to Sanden et al. [29], wooden breast had more diffuse and broader connective tissues with more gaps, showing a thin and thick mixture of collagen fibers, and IMCT denaturation studied by DSC showed the presence of different endothermic peaks in the range of 50–80 °C [30]. As for the surface of the *pectoralis major* muscle, the total enthalpy of protein denaturation was found to be significantly lower (*p* < 0.05) in the WB group if compared to the NB group (3.2 vs. 3.92 J/g) [31,32]. Collagen biosynthesis and intermolecular crosslinking is a complex biological process mediated by a series of key regulators [33]. More collagen cross-linking decreases the elasticity of collagen fibers, leading to increased tissue stiffness and reduced meat quality, with lower ΔH. Although an increase in the amount of connective tissue components was found in the WB case mentioned above, thermally inert cross-linking could be responsible for a similar evolution of compression and shear force values measured on WB and NB cooked samples [31]. It is well known that in skeletal muscle there are three layers of connective tissue containing extracellular matrix macromolecules, including epimysium, perimysium and endomysium. The predominant extracellular matrix proteins in these layers are fibrillar collagens, particularly types I and III [34]. Although different SDS-PAGE patterns were found, it is not very clear how these collagen types are affected by WB, if both type I and type III collagens were affected in the current study. This warrants more research in the future. Notably, the results showed that the two fractions of myofibrillar proteins and sarcoplasmic proteins are probably being replaced by connective tissue, thus contributing to increased muscle hardness.

### 3.4. Protein Extraction Characteristics

**Figure 5:**
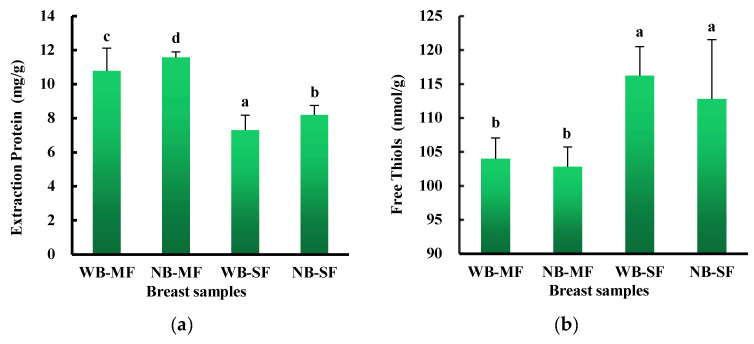
Protein extraction content (**a**) and free thiol content (**b**) in wooden breast (WB) and normal breast (NB) muscles of broilers in myofibrillar (MF) and sarcoplasmic (SF) protein fractions. Means within groups without a match in superscripts (abcd) differ, *p* < 0.05.

Our results also showed that when protein was extracted from 1d postmortem samples, the sarcoplasmic protein fraction of WB had significantly lower protein content compared to NB (Figure 5a). In addition, the protein content of the myofibrillar protein fraction was also significantly lower in WB compared to NB (*p* < 0.05). However, the current study showed a trend towards increased thiol content in both the myofibrillar protein fraction and the sarcoplasmic protein fraction as between WB and normal individuals, although no significant differences were found (Figure 3b). Li et al. [35] and Carvalho et al. [36] reported greater loss of carbonyl content and free thiol groups in severe wooden breast samples (*p* < 0.05). In this study, more subtle differences arose between WB and NB, which may contribute to altering the oxidative homeostasis associated with increased oxidative stress in severe WB muscle [37]. In general, the loss of free thiols indicates the formation of disulfide cross-linked myosin heavy chains [38]. It is also important to mention that the WB samples exhibited a significantly higher level of protein carbonyls (*p* < 0.05) indicating that a greater degree of protein oxidation was found [39]. As also mentioned above, more intramuscular connective tissue could be extracted after blending from WB samples compared to NB samples, not only at day 1 but also at day 3 postmortem. In sum, in our study, WB samples displayed worse raw meat texture characteristics on 1 day postmortem compared to 3 days postmortem. Notably, the differences among the groups were mainly detected when raw meat rather than cooked was analyzed. However, higher cook loss and lower shear force of WB compared with NB were found after prolonged 4 °C storage [12,40].

In conclusion, this investigation suggests that the presence of WB has adverse effects on meat quality characteristics such as raw meat hardness and lower water retention capacity. WB has a higher collagen content, including insoluble collagen, which may help explain the hardness of its raw meat. The different thermal properties of isolated IMCT may be explained by the different protein composition in the wooden pectoral muscle. These results could provide more information on the meat processing properties of wooden pectoral muscle and could serve as a guide for the future. Therefore, future studies should elucidate the links that exist between the types of collagen and their involvement in the development of meat texture and thermal properties, in order to better understand the mechanisms underlying the condition of wooden breast myopathy.

## Figures and Tables

**Table 1:** Thermal properties of intramuscular connective tissue from wooden breast (WB) and normal breast (NB) muscles at day 1 and day 3 postmortem shown as onset, peak and endset denaturation temperatures, and the denaturation enthalpy (ΔH).

	WB-1d	NB-1d	WB-3d	NB-3d
onset T (°C)	58.22 ± 0.82 ^a^	57.24 ± 2.77 ^a^	56.70 ± 0.76 ^a^	57.66 ± 2.33 ^a^
peak T (°C)	63.73 ± 0.36 ^a^	63.58 ± 2.17 ^a^	62.81 ± 0.25 ^a^	64.04 ± 1.87 ^a^
endset T (°C)	71.55 ± 0.66 ^a^	72.62 ± 1.59 ^a^	69.91 ± 0.90 ^b^	72.84 ± 1.13 ^a^
ΔH (J/g)	9.76 ± 4.34 ^a^	14.71 ± 5.04 ^b^	7.86 ± 3.69 ^a^	14.16 ± 3.62 ^b^

Each treatment was performed in triplicate (*n* = 6). Means within rows having different superscripts (ab) differ, *p* < 0.05.

## Data Availability

The datasets generated for this study are available on request to the corresponding author.
